# ScAlN PMUTs Based on Flexurally Suspended Membrane for Long-Range Detection

**DOI:** 10.3390/mi15111377

**Published:** 2024-11-14

**Authors:** Shutao Yao, Wenling Shang, Guifeng Ta, Jinyan Tao, Haojie Liu, Xiangyong Zhao, Jianhe Liu, Bin Miao, Jiadong Li

**Affiliations:** 1 Key Laboratory of Multifunctional Nanomaterials and Smart Systems, Suzhou Institute of Nano-Tech and Nano-Bionics, Chinese Academy of Sciences, Suzhou 215123, China; styao2023@sinano.ac.cn (S.Y.); wlshang2021@sinano.ac.cn (W.S.); jytao2023@sinano.ac.cn (J.T.); 2 School of Electrical and Mechanical Engineering, Changchun University of Science and Technology, Changchun 130022, China; gfta2023@sinano.ac.cn (G.T.); hjliu2023@sinano.ac.cn (H.L.); a_liu100@163.com (J.L.); 3 School of Nano-Tech and Nano-Bionics, University of Science and Technology of China, Hefei 230026, China; 4 Key Laboratory of Optoelectronic Material and Device, Department of Physics, Shanghai Normal University, Shanghai 200234, China; xyzhao@shnu.edu.cn

**Keywords:** piezoelectric micromachined ultrasonic transducer (PMUT), scandium-doped aluminum nitride (ScAlN), flexurally suspended membrane, long-range target detection

## Abstract

Piezoelectric micromachined ultrasonic transducers (PMUTs) have been widely applied in distance sensing applications. However, the rapid movement of miniature robots in complex environments necessitates higher ranging capabilities from sensors, making the enhancement of PMUT sensing distance critically important. In this paper, a scandium-doped aluminum nitride (ScAlN) PMUT based on a flexurally suspended membrane is proposed. Unlike the traditional fully clamped design, the PMUT incorporates a partially clamped membrane, thereby extending the vibration displacement and enhancing the output sound pressure. Experimental results demonstrate that at a resonant frequency of 78 kHz, a single PMUT generates a sound pressure level (SPL) of 112.2 dB at a distance of 10 mm and achieves a high receiving sensitivity of 12.3 mV/Pa. Distance testing reveals that a single PMUT equipped with a horn can achieve a record-breaking distance sensing range of 11.2 m when used alongside a device capable of simultaneously transmitting and receiving ultrasound signals. This achievement is significant for miniaturized and integrated applications that utilize ultrasound for long-range target detection.

## 1. Introduction

Ultrasonic transducers are devices capable of converting electrical energy into mechanical energy and vice versa. They are widely utilized in distance measurement applications, such as parking assistance [1,2], industrial auxiliary measurement [3,4], and robot obstacle avoidance [5,6]. Piezoelectric micromachined ultrasonic transducers (PMUTs), which are based on microelectromechanical system (MEMS) technology, offer distinct advantages over traditional bulk piezoelectric ultrasonic transducers, including compact size, low power consumption, and ease of integration [7,8,9,10]. As a result, PMUT can be integrated into new portable products, such as wearable devices and smart home products, thereby expanding the application scope of these systems.

The piezoelectric materials of PMUT include polyvinylidene fluoride (PVDF) [11], lead zirconate titanate (PZT) [12], zinc oxide (ZnO) [13], and aluminum nitride (AlN) [14]. Among them, PZT and AlN are widely used in distance detection applications. PZT exhibits a notable piezoelectric coefficient, resulting in high transmission sensitivity; however, its application range is limited by factors such as high deposition temperature and challenges associated with integration. In contrast, although AlN possesses a lower piezoelectric coefficient compared to PZT, it is regarded as capable of achieving comparable performance due to its lower dielectric constant relative to that of PZT. AlN exhibits a great receiving sensitivity, a low deposition temperature, and excellent compatibility with complementary metal oxide semiconductor (CMOS) fabrication processes [15,16,17]. Moreover, doping scandium (Sc) into AlN (Sc_x_Al_1−x_N) not only maintains the exceptional reception performance of AlN but also enhances its transmission characteristics [18,19,20]. Consequently, PMUT based on AlN, particularly ScAlN, has great potential for miniaturized and integrated ranging applications.

In recent years, AlN PMUT for distance detection has made remarkable progress [21,22,23,24,25], and the maximum measurement distance of the PMUT has been increased to 9 m [25]. The current level of advancement has been able to meet the needs of most applications. However, in fields such as intelligent robotics, the requirements for ranging sensors become even more stringent, particularly for micro-robots operating in complex and dynamic environments [26,27]. These micro-robots require the ability to accurately perceive obstacles at considerable distances while moving at high speeds (potentially exceeding 10 m per second) to ensure operational efficiency and safety. The reported maximum measurement distance of PMUT cannot meet such requirements, so it is particularly important to further improve its measurement performance.

In this study, we designed, fabricated, and characterized a flexurally suspended PMUT based on an SOI wafer, which is formed by sputtering the bottom electrode and Sc_0.2_Al_0.8_N film on a composite film of Si and SiO_2_. This structure can significantly enhance both vibration displacement and output pressure. A single PMUT generates a sound pressure level (SPL) as high as 112.2 dB at a distance of 10 mm and achieves a high receiving sensitivity of 12.3 mV/Pa. Furthermore, the measurement performance of the PMUT was evaluated with distance testing experiments, successfully achieving a sensing distance of 11.2 m, the farthest sensing distance recorded to date. These advancements are of significant importance for miniaturized and integrated mobile ranging applications.

## 2. PMUT Design and Modeling

### 2.1. Structural Design

The studied PMUT is composed of three material layers in the order Mo/ScAlN/Au grown sequentially on an SOI wafer. The electric field is generated by applying a voltage between the upper and lower electrodes of the PMUT, which leads to the creation of lateral stress in the piezoelectric layer. This stress causes the circular film to bend outward, resulting in the generation of a pressure wave. Similarly, when an incident pressure wave is encountered, it causes deformation in the film, utilizing the piezoelectric effect to generate charges on the electrodes [28]. These charges enable the device to transmit and receive ultrasound signals.

**Figure 1:**
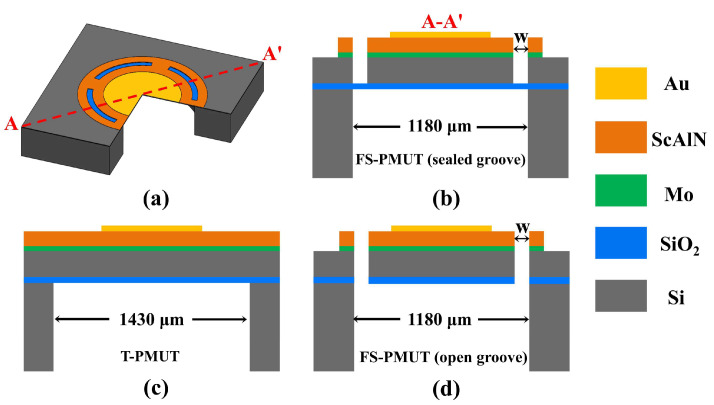
(**a**) Structure of the PMUT; (**b**–**d**) cross-sectional structure of FS-PMUT with sealed grooves, T-PMUT, and FS-PMUT with open grooves.

To increase the device output pressure, we selected a Sc_0.2_Al_0.8_N film, which has a higher piezoelectric coefficient than AlN films [29,30], as the piezoelectric material. Structurally, we adopted a flexurally suspended membrane supported by four flexures and a thin SiO_2_ layer to extend the vibration displacement and improve acoustic coupling. The proposed ScAlN PMUT based on the flexurally suspended membrane (FS-PMUT) is formed by etching four grooves, as shown in Figure 1a,b. Compared to the traditional clamping PMUT (T-PMUT, Figure 1c), stiffness modification can be achieved by etching grooves on the surface of the PMUT, thus enabling the residual stress to be localized and enhancing the transmission sensitivity [31,32]. Additionally, compared to the FS-PMUT with open grooves (Figure 1d), the sealed groove design can improve acoustic characteristics.

**Figure 2:**
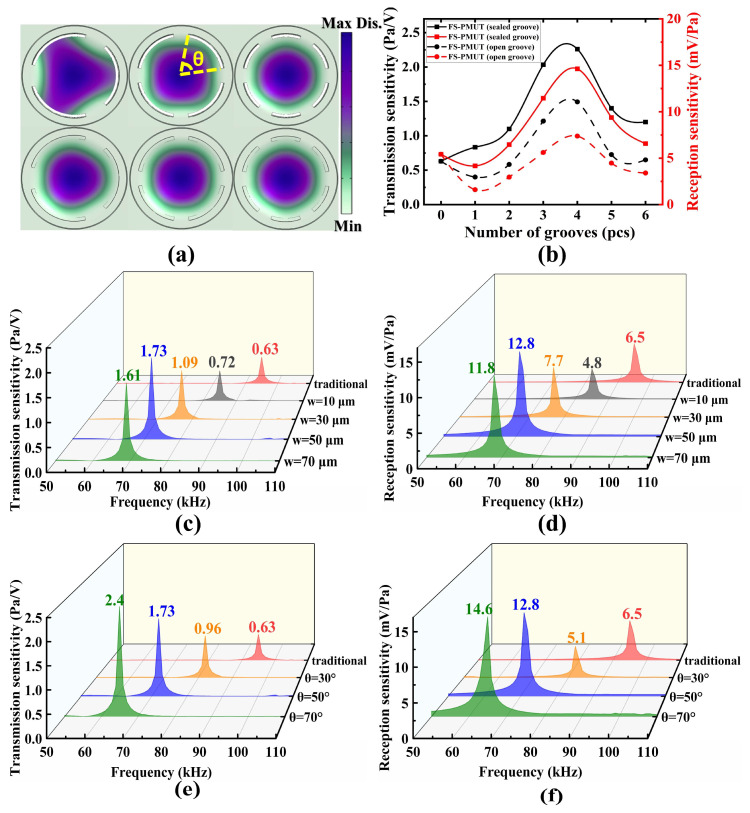
(**a**) The first-order modal shapes of FS-PMUT with different numbers of grooves, including those with open grooves (top) and those with sealed grooves (bottom); (**b**) transmission sensitivity and reception sensitivity of FS-PMUT with different numbers of grooves, including those with open grooves (dashed line) and with sealed grooves (solid line); (**c**,**d**) transmission and reception sensitivity of FS-PMUT with four sealed grooves of different widths; (**e**,**f**) transmission and reception sensitivity of FS-PMUT with four sealed grooves of different angles.

The optimization of the FS-PMUT dimensional using COMSOL Multiphysics 6.0 is essential for enhancing performance. To maximize the vibration displacement of the membrane, we set the depth of the grooves to be the sum of the thicknesses of the piezoelectric layer and the structural layer. With the groove depth determined, our design considerations included the number and size of the grooves, as well as whether the grooves are sealed or not. Figure 2a illustrates the first-order modal shapes of PMUTs with different types of grooves. It can be seen that the sealed groove design has little effect on the mode shape, these modal shapes exhibit a consistent trend, the center amplitude was the largest, and the edge amplitude was the smallest. And it appears that the PMUT with four grooves has a larger vibration area. Figure 2b shows the transmission and reception sensitivity at a distance of 10 mm from a single FS-PMUT. The results show that the PMUT with four grooves has better acoustic characteristics. Furthermore, the findings suggest that the design with open grooves results in unsatisfactory acoustic characteristics. Therefore, we chose the design of FS-PMUT with four sealed grooves.

Figure 2c–f illustrates the impacts of different groove widths (w) and angles (θ) on the acoustic characterization of FS-PMUT with four sealed grooves. In this work, we initially set the groove angle at 50° and determined the optimal groove width to be 50 μm (Figure 2c,d). Subsequently, with a groove width of 50 μm established, we identified the optimal groove angle as 70° (Figure 2e,f).

### 2.2. Equivalent Circuit Model

**Figure 3:**
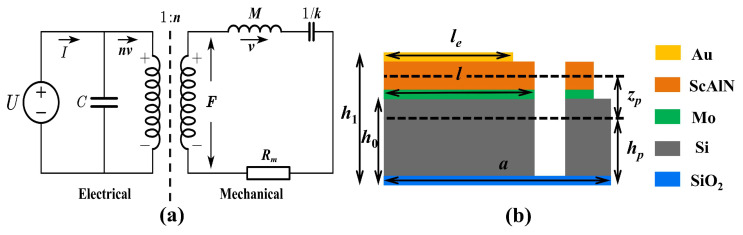
(**a**) Equivalent circuit model, including the electrical domain (left) and mechanical domain (right); (**b**) schematic diagram of the boundary structure.

PMUTs are electromechanical devices that convert mechanical energy into electrical energy and vice versa by coupling transverse stresses with electric fields in piezoelectric materials [33]. Therefore, their operation can be simulated using an equivalent circuit model comprising electrical and mechanical domains (Figure 3a). The governing equations of the equivalent circuit model are as follows [34]:

(1) I=nv+jωCU,

(2) $$F=j\omega Mv+\frac{kv}{j\omega }+R_{m}v.$$

The frequency response of PMUT can be analyzed through examination of its equivalent circuit model, where U is the input voltage, I is the current, C is the parallel capacitance, F is the mechanical force, M is the mechanical mass, k is the mechanical stiffness, $R_{m}$ is the mechanical impedance, v is the velocity, ω is the frequency, and n is electromechanical conversion factor.

The proposed FS-PMUT utilizes four grooves to reduce the film stiffness, which lead to increases in both the center vibration displacement ($x_{0}$) and effective vibration area ($A_{\mathrm{effective}}$) of the film, where $x_{0}$ and $A_{\mathrm{effective}}$ are defined by the following equations:

(3) $$x_{0}=\frac{x(r)}{\varphi (r)},$$

(4) $$A_{\mathrm{effective}}=2\;\pi \int_{0}^{a}r\varphi (r)dr.$$

The mode shapes of FS-PMUT and T-PMUT are obtained using the polynomial curve fitting function based on the simulated data of the vibration modes, as described below:

(5) $$\varphi_{\mathrm{FS}}(r)=1-1.271{\left(\frac{r}{a}\right)}^{2}+0.3634{\left(\frac{r}{a}\right)}^{4},$$

(6) $$\varphi_{T}(r)={\left(1-{\left(\frac{r}{a}\right)}^{2}\right)}^{2},$$

where r is the distance from the center of the membrane, and a is the radius of the diaphragm. Figure 3b depicts the boundary schematic of the FS-PMUT. Here, $h_{0}$ is the total thickness of the structural support layers (Si and SiO_2_), $h_{1}$ is the total structural thickness, $h_{p}$ is the distance from the bottom layer to the neutral axis, and $z_{p}$ is the distance between the center of the piezoelectric layer and the neutral axis.

The effective vibration areas of the FS-PMUT ($A_{\mathrm{FS}}$) and the T-PMUT ($A_{T}$) can be calculated using Equation (4): $A_{\mathrm{FS}}=0.486\;\pi a^{2};$ $A_{T}=0.33\;\pi a^{2}.$ Consequently, the effective vibration area of the FS-PMUT increases by a factor of 1.47. The formulas for the mechanical stiffness and mechanical mass are derived based on the principles of the conservation of elastic energy and kinetic energy [35]:

(7) $$k=4\;\pi \int Y_{0}^{\prime \prime }z^{2}dz\int_{0}^{a}r[{\left(\frac{\partial^{2}\varphi }{\partial r^{2}}\right)}^{2}+\frac{2\upsilon }{r}\frac{\partial^{2}\varphi }{\partial r^{2}}\frac{\partial \varphi }{\partial r}+{\left(\frac{1}{r}\frac{\partial \varphi }{\partial r}\right)}^{2}]dr,$$

(8) $$M=2\;\pi \mu \int_{0}^{a}r\varphi^{2}(r)dr,$$

where $Y_{0}^{\prime \prime }$ is the effective value of Young’s modulus, υ is the effective value of Poisson’s ratio, z is the distance from the neutral axis, and μ is area mass density. The bending stiffness D $\left(D=\int Y_{0}^{\prime \prime }z^{2}dz\right)$ of the system is evaluated based on the parameters of each part (Figure 3b) and can be used to obtain the stiffness and mass $(k_{\mathrm{FS}}\approx \frac{14.58\;\pi D}{a^{2}},M_{\mathrm{FS}}\approx 0.295\;\mu \pi a^{2};$ $k_{T}\approx \frac{21.3\;\pi D}{a^{2}},M_{T}\approx 0.2\;\mu \pi a^{2}).$ According to the formula for resonance frequency [36], we derived the first-order resonance frequency equations for FS-PMUT and T-PMUT:

(9) $$f_{\mathrm{FS}}\approx \frac{7.03}{2\;\pi a^{2}}\sqrt{\frac{D}{\mu }};f_{T}\approx \frac{10.32}{2\;\pi a^{2}}\sqrt{\frac{D}{\mu }}.$$

The electromechanical coupling energy is subsequently utilized to derive the electromechanical conversion factor [33], which can be calculated by the following equations:

(10) $$W_{\mathrm{coupl}}=z_{p}e_{31,f}I_{\mathrm{piezo}}Ux_{0},$$

(11) $$I_{\mathrm{piezo}}=2\;\pi \int r(\frac{\partial^{2}\varphi }{\partial r^{2}}+\frac{1}{r}\frac{\partial \varphi }{\partial r})dr,$$

(12) $$n=2\;\pi z_{p}e_{31,f}\int r(\frac{\partial^{2}\varphi }{\partial r^{2}}+\frac{1}{r}\frac{\partial \varphi }{\partial r})dr,$$

where $e_{31,f}$ is the piezoelectric constant, and $I_{\mathrm{piezo}}$ is the strain integral in the electrode region. Thus, the vibration displacements $x_{dc}$ $\left(x_{dc}=\frac{nU}{k}\right)$ can be obtained according to Equations (7) and (12) as follows:

(13) $$x_{\mathrm{FS\_dc}}\approx \frac{2.57a^{2}z_{p}e_{31,f}}{Y_{0}^{\prime \prime }h_{p}^{3}};x_{T\_dc}\approx \frac{1.1a^{2}z_{p}e_{31,f}}{Y_{0}^{\prime \prime }h_{p}^{3}}.$$

The results indicate that the effective vibration area of the FS-PMUT increases by a factor of 1.47, and the vibration displacement increases by more than two times.

## 3. Analysis Verification

In order to normalize the effects of resonance frequency on PMUT characteristics, the radius of the designed FS-PMUT was 590 μm, while that of the T-PMUT was increased to 715 μm. Finite element modelling was established using the acoustic–piezoelectric interaction module in COMSOL Multiphysics to verify the characteristics of the three PMUTs at similar frequencies. The structural parameters for both FS-PMUT and T-PMUT are presented in Table 1. The necessary material parameters for the PMUT model can be accessed within the COMSOL Material Depot. During the modeling process, it is imperative to apply constraints to the bottom boundary of the base substrate. The electrostatic boundary condition involved setting a voltage of 10 Vpp at the top electrode, while the bottom electrode was grounded. When meshing the grid for this PMUT model, the maximum dimension should not exceed one fifth of the wavelength corresponding to the specified material.

**Figure 4:**
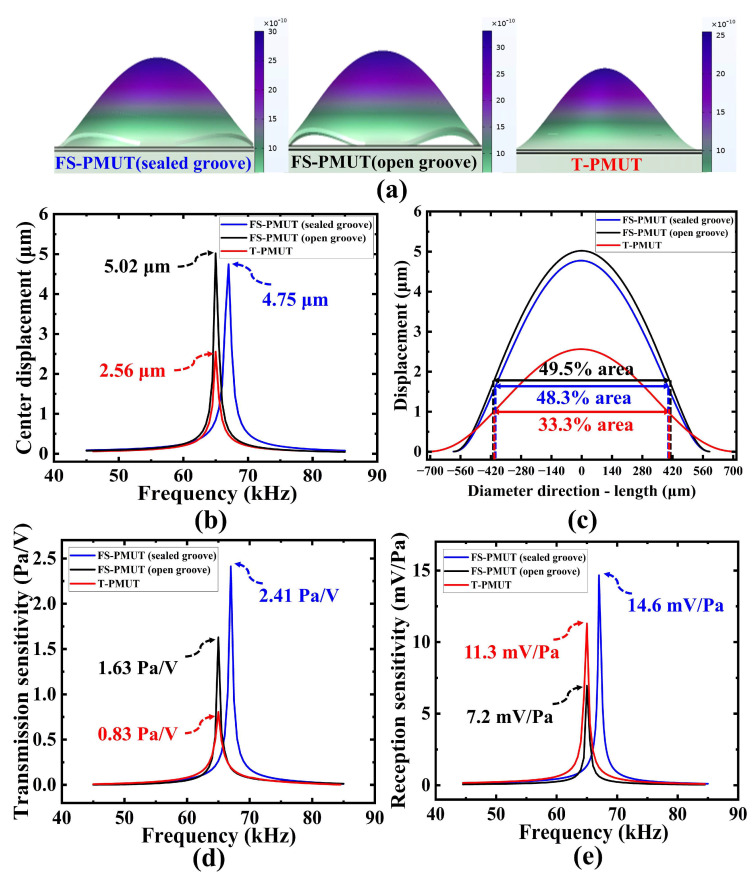
Simulated frequency responses of FS-PMUT and T-PMUT at their resonance frequencies: (**a**) mode shapes of FS-PMUT and T-PMUT; (**b**) center displacement of PMUT at 10 Vpp; (**c**) comparison of vibration displacement along the diameter direction of the PMUT. (**d**) Transmission sensitivity at a distance of 10 mm; (**e**) comparison of reception sensitivity.

Figure 4 shows the frequency response of the PMUT driven by 10 Vpp (peak-to-peak) voltage. Figure 4a compares the mode shapes of the three PMUTs at their resonance frequencies, revealing that the FS-PMUT is released at its edges and has a large vibration area and displacement. Figure 4b shows the displacement at the center point of the PMUT. The results show that this is 4.75 μm for the FS-PMUT with sealed grooves, 5.02 μm for the FS-PMUT with open grooves, and 2.56 μm for the T-PMUT, indicating a twofold increase in the center displacement. Figure 4c illustrates the vibration displacement along the direction of the film diameter at the resonant frequency. Taking the −9 dB as a reference, the effective vibration area of the FS-PMUT with sealed grooves is 48.3% of the total area, that of the FS-PMUT with open grooves is 49.5%, and that of the T-PMUT is 33.3%, indicating a 1.5-fold increase in the effective vibration area.

Figure 4d presents the transmission sensitivity simulation data at a distance of 10 mm from the single PMUT. The results show that the transmission sensitivity of the FS-PMUT with sealed grooves is 1.5 times greater than that of the FS-PMUT with open grooves, and nearly three times greater than that of the T-PMUT at a similar frequency. In Figure 4e, the reception sensitivity of a single PMUT is compared. An acoustic pressure of 1 Pa is applied at a distance of 10 mm from the PMUT, after which its output voltage response is evaluated. The results demonstrate that the FS-PMUT with sealed grooves exhibits better reception sensitivity than others.

In addition, the results show that the FS-PMUT with open grooves does not have outstanding acoustic characteristics, although it has a large vibration displacement. It could potentially be attributed to the ventilation of two sides of the membrane [31]. In contrast, the FS-PMUT with sealed grooves has both enhanced vibration displacement and output pressure. It further validates that the sealed groove design can improve acoustic coupling, aligning with the results presented in Figure 2b.

## 4. Materials and Methods

**Figure 5:**
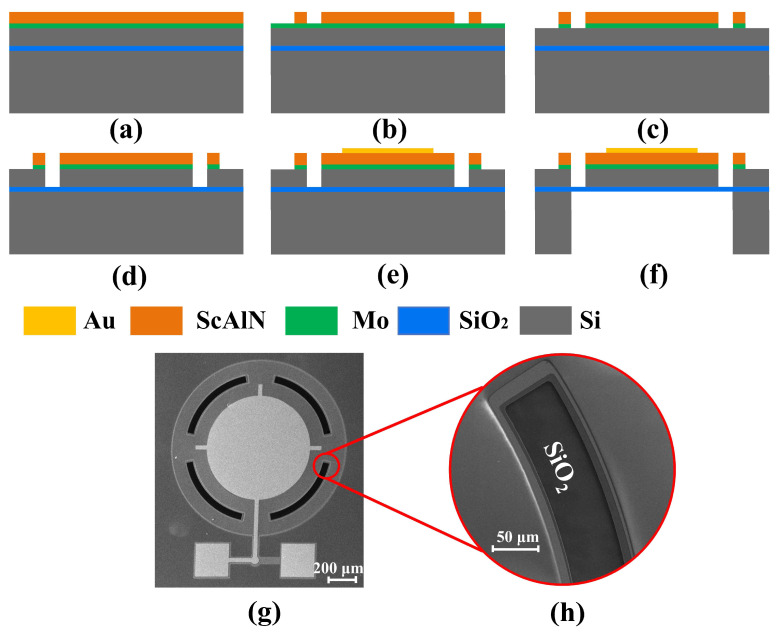
(**a**–**f**) FS-PMUT (sealed groove) fabrication process flow; (**g**,**h**) scanning electron microscopy of the upper surface of FS-PMUT (sealed groove).

The process flow of the FS-PMUT (Figure 5) began with a ScAlN/Mo/SOI wafer, where the thickness of ScAlN was 1 µm, the thickness of Mo was 0.2 µm, and the SOI wafer comprised a 5 µm device silicon layer, a 0.2 µm buried oxide layer, and a silicon substrate (Figure 5a). The ScAlN/Mo/SOI wafer was provided by Shanghai Normal University, Shanghai, China. Initially, the ScAlN film was patterned using ion beam etching (Figure 5b). Next, plasma etching was employed to pattern the bottom electrode Mo layer (Figure 5c). Subsequently, deep reactive ion etching was utilized to etch the support layer silicon (Figure 5d). Following this etching step, a portion of the wafer surface was protected by a photoresist, while another part underwent plasma etching treatment on exposed SiO_2_ patterns to produce two types of FS-PMUT: one with sealed grooves and one with open grooves. Then, 200 nm of gold was sputtered above the piezoelectric material as the top electrode (Figure 5e). Finally, the backside of the wafer was etched by deep reactive ion etching (DRIE), which stops at the SiO_2_ layer (Figure 5f). The final fabricated FS-PMUT, with a radius of 590 μm and sealed grooves, is depicted in Figure 5g. In this work, a T-PMUT was fabricated for experimental comparison purposes; the corresponding test results are presented in the subsequent section.

## 5. Results and Discussion

**Figure 6:**
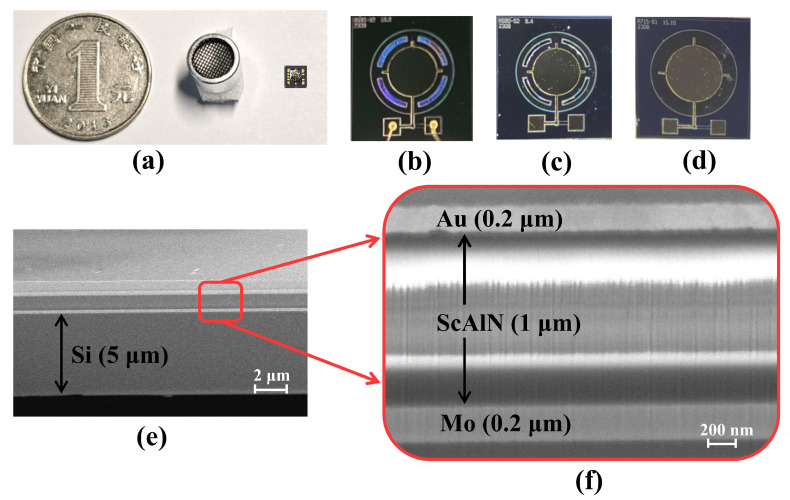
(**a**) Device dimensions (2 mm × 2 mm × 0.3 mm) relative to a coin and a conventional ultrasound transducer; (**b**–**d**) the optical images of the three PMUT structures mentioned in this work: (**b**) is FS-PMUT with sealed grooves, (**c**) is FS-PMUT with open grooves, (**d**) is T-PMUT; (**e**,**f**) the cross-sectional view taken through a scanning electron microscope.

Figure 6a depicts the device dimensions relative to a coin and a conventional ultrasound transducer. The entire chip has lateral dimensions of 2 mm × 2 mm and a thickness of 0.3 mm. Figure 6b–d show optical images of the FS-PMUT, with a radius of 590 μm, and the T-PMUT, with a radius of 715 μm. In Figure 6e, a cross-sectional image captured by scanning electron microscopy reveals that the materials were deposited under good conditions. To comprehensively evaluate the characteristics of the three PMUT structures mentioned above, we examined their characteristics from three perspectives: mechanical, electrical, and acoustic characteristics.

### 5.1. Mechanical Characterization

**Figure 7:**
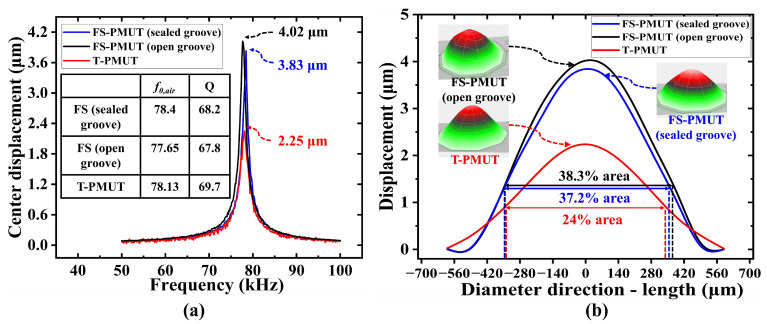
LDV test results of FS-PMUT (R590) and T-PMUT (R715). (**a**) center displacement of PMUT at 10 Vpp; (**b**) the displacement along the diameter direction of the PMUT.

The mechanical characteristics of PMUTs were measured by a laser Doppler vibrometer (LDV). Figure 7a shows the displacement at the center point of the PMUT at 10 Vpp. The results show that the peak displacements are 4.02 μm for FS-PMUT (open groove), 3.83 μm for FS-PMUT (sealed groove), and 2.25 μm for T-PMUT. They signify a 1.8-fold increase in the FS-PMUT vibration displacement at the same frequency, which closely aligns with the 2-fold increase observed in the simulation results.

The calculated quality factors (Q) of the FS-PMUT with open grooves is 68.2, that of the FS-PMUT with sealed grooves is 67.8, and that of the T-PMUT is 69.7. The results show that there is no significant difference in Q-factor. Figure 7b illustrates the vibration displacement over the same range along the direction of diameter at resonant frequency. Taking −9 dB as a reference, the effective vibration area of the FS-PMUT with sealed grooves is 37.2% of the total area, that of the FS-PMUT with open grooves is 38.3%, and that of the T-PMUT is 24%. This means that the effective vibration area of FS-PMUT increased by 1.5 times, which is consistent with the simulation results.

Additionally, the actual frequency of our device ranges from 77 kHz to 79 kHz. The discrepancies between experimental and simulated results can be attributed to several factors: the simulations assume ideal system boundary conditions, including material thickness, diameter, and properties. In contrast, fabrication introduces variability such as thickness errors, residual stress, and changes in film diameter and thickness due to incomplete etching.

### 5.2. Electrical Characterization

**Figure 8:**
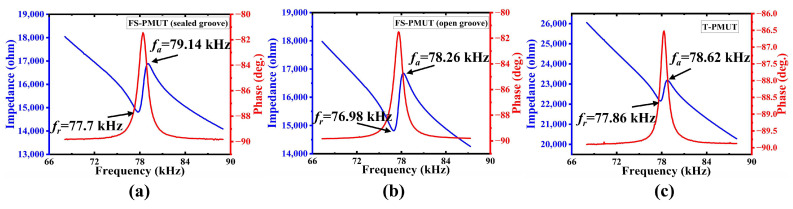
Measured electrical impedance values of a single PMUT in air. (**a**) electrical characteristics of FS-PMUT with sealed grooves; (**b**) electrical characteristics of FS-PMUT with open grooves; (**c**) electrical characteristics of T-PMUT.

The electrical characteristics of the PMUT were assessed using a precision impedance analyzer (MICRO-TEST 6632, Suzhou, China), as shown in Figure 8. The effective coupling coefficient can be determined using the following equation [37]:

(14) $$k_{\mathrm{eff}}^{2}=\frac{\pi^{2}}{8}\frac{f_{a}^{2}-f_{r}^{2}}{f_{a}^{2}}=\frac{C_{m}}{C_{m}+C_{0}},$$

where $f_{r}$ and $f_{a}$ are the series and parallel resonant frequencies, respectively; $C_{m}$ is the motional capacitance; and $C_{0}$ is the static capacitance, which is 122.97 pF for the FS-PMUT with sealed grooves, resulting in a $k_{\mathrm{eff}}^{2}$ of 4.45% (Figure 8a). The static capacitance is 125.86 pF for the FS-PMUT with open grooves, resulting in a $k_{\mathrm{eff}}^{2}$ of 4% (Figure 8b), while it is 94.06 pF for the T-PMUT, resulting in a $k_{\mathrm{eff}}^{2}$ of 2.37% (Figure 8c). This means a nearly twofold increase in the effective coupling coefficient of the FS-PMUT. Moreover, it is 233% greater than that of the AlN PMUT reported in [38] and approximately 123% greater than that of the Sc_0.2_Al_0.8_N PMUT reported in [23]. The combination of scandium doping and the flexurally suspended membrane structure enhances $k_{\mathrm{eff}}^{2}$ and makes the energy conversion efficiency higher.

### 5.3. Acoustic Characterization

The output pressure from a single PMUT is measured by a calibrated reference microphone (Gras 46BF-1, Holte, Denmark). The measurement is taken at 10 mm via continuous wave driving at 14.7 Vpp and at the resonant frequency. The output pressure of the FS-PMUT with sealed grooves is 8.15 Pa (112.2 dB SPL), that of the FS-PMUT with open grooves is 4.7 Pa (107.4 dB SPL), and that of the T-PMUT is 2.76 Pa (102.8 dB SPL) (temperature: 23 °C, relative humidity: 78%). The results indicate that the output pressure of the FS-PMUT with sealed grooves is nearly three times greater than that of the T-PMUT and 1.5 times greater than that of the FS-PMUT with open grooves. Comparing the measured results with the simulation results, the measured output pressure is less than half of the simulation result. This may be due to the reduction in vibration displacement and vibration area.

**Figure 9:**
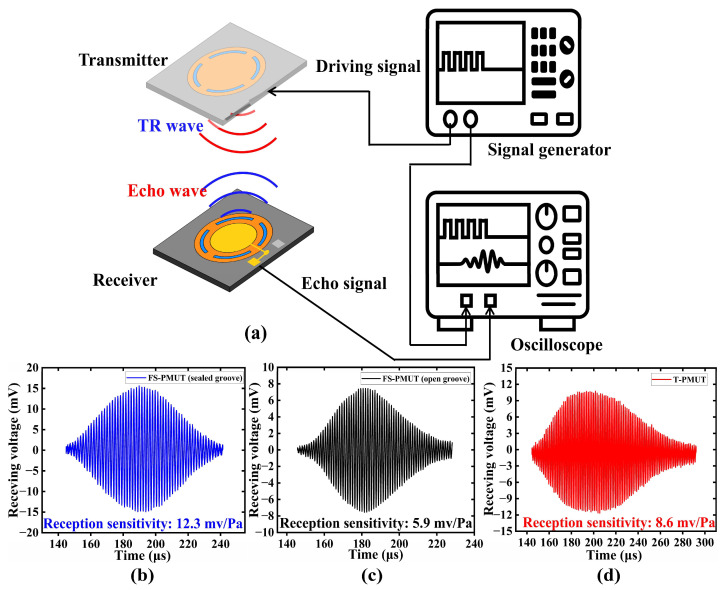
(**a**) Schematic diagram of experimental set up; (**b**–**d**) comparison results of the reception sensitivity.

In addition, the reception sensitivity of the PMUT was also tested. Initially, we calibrated the PMUT transmitters using reference microphones. According to LDV test results, the resonant frequencies of the PMUTs are 78.4 kHz (FS-PMUT with sealed grooves), 77.65 kHz (FS-PMUT with open grooves), and 78.13 kHz (T-PMUT). Subsequently, reception sensitivity tests were conducted for the PMUT at their respective frequencies. We excited transmitters at these frequencies with a continuous wave, resulting in an output pressure of 96 dB (SPL) at about 50 mm. A schematic diagram of the experimental set up is shown in Figure 9a. The experimental results are shown in Figure 9b–d, in which the sensitivity of FS-PMUT with sealed grooves is 12.3 mV/Pa, that of the FS-PMUT with open grooves is 5.9 mV/Pa, and that of the T-PMUT is 8.6 mv/Pa. This indicates that the reception sensitivity of the FS-PMUT with sealed grooves is two times that of the FS-PMUT with open grooves and 1.5 times that of the T-PMUT.

Table 2 presents the test results outlining the mechanical characteristics, electrical characteristics, and acoustic characteristics of the three different PMUT designs. The results show that the FS-PMUT with sealed grooves exhibits superior performance compared with the T-PMUT at similar resonant frequencies. Furthermore, the smaller size of the FS-PMUT at identical frequencies contributes to a reduced transducer volume and increases the fill factor in an array configuration.

## 6. Rangefinding Experiments

**Figure 10:**
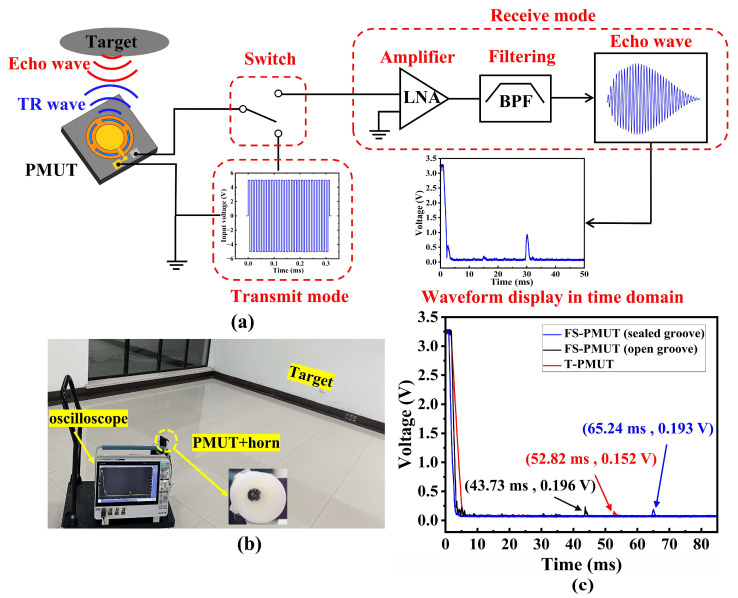
(**a**) The block diagram of the system; (**b**) experimental assembly for distance testing; (**c**) envelope curves of the echo signal during distance measurement using a single PMUT.

The performance of the PMUT was validated via a distance testing experiment employing a system capable of simultaneously transmitting and receiving ultrasonic signals. The block diagram of the system is shown in Figure 10a. When the system is in transmit mode, it sends out a 15 Vpp, 78 kHz square wave signal consisting of 30 cycles to excite a single PMUT, which generates ultrasonic waves. Upon the completion of the transmission, the system transitions to reception mode using a transmit–receive isolation switch. The ultrasonic waves are reflected by the detection target, and the echoes are received by the single PMUT. Then, the received echo signal is converted into a voltage signal. The low noise amplifier (LNA) amplifies the signal, and a bandpass filter (BPF) then filters the amplified out-of-band noise. Finally, the returned signal is displayed as an envelope on the oscilloscope. The distance from the device to the detection target can be calculated based on the time corresponding to the amplitude of the maximum echo signal [39].

The experimental assembly for distance testing is depicted in Figure 10b. The PMUT was connected to a PCB mounted on a mobile system. The system was powered by an external power, and the ultrasonic echo signal was displayed as an envelope on the oscilloscope. In this work, we added the same horn separately to a single PMUT of three different structures to concentrate the output pressure [24,40]. In addition, different ambient temperatures and humidity levels will lead to different ultrasonic transmission losses in the air [39]. In this work, the temperature of this ranging experiment was 15 °C and the relative humidity was 37%. Figure 10c shows the results obtained by long-range detection of three PMUTs under the same conditions. Specifically, the FS-PMUT with sealed grooves (blue lines) detected a signal amplitude of 193 mV at 65.24 ms. Using the ultrasonic time-of-flight (TOF) principle, the results show that it achieved a sensing distance of about 11.2 m, the largest detection distance of the three PMUT configurations when compared. The distance detection results are consistent with the performance characteristics reported in the previous chapter.

**Figure 11:**
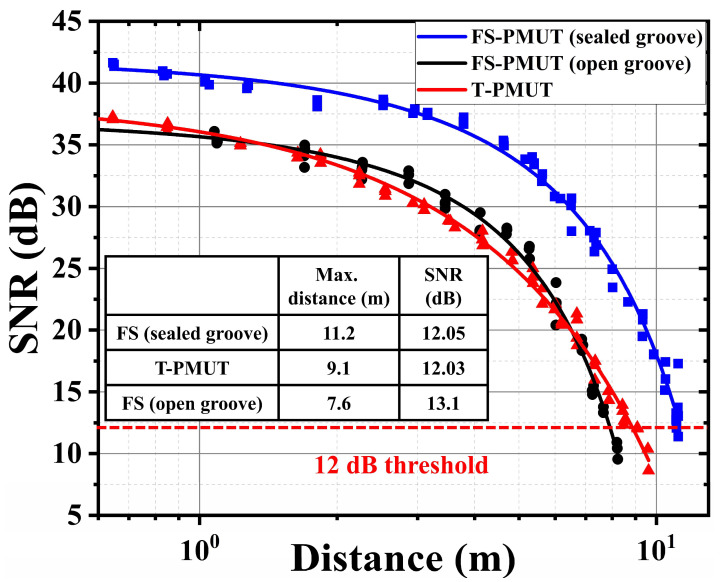
SNR versus distance: at a threshold of 12 dB, the maximum range of the FS-PMUT with sealed grooves is 11.2 m.

Figure 11 summarizes the signal-to-noise ratios (*SNR*) for the distance test results of the FS-PMUT and T-PMUT. The *SNR* is calculated as follows:

(15) $$SNR=20\;\log_{10}(\frac{\Delta V_{\mathrm{signal}}}{\Delta V_{\mathrm{noise}}}),$$

where $\Delta V_{\mathrm{signal}}$ is the difference between the return signal voltage amplitude and the system base value, and $\Delta V_{\mathrm{noise}}$ is the difference between the system noise amplitude and the system base value. The determination of the maximum measurement range heavily relies on achieving a sufficient SNR. To mitigate the occurrence of false signals, we adopt the threshold for this minimum SNR as mentioned in [41]. Therefore, the results presented in Figure 11 demonstrate that a single FS-PMUT with sealed grooves driven at 78 kHz achieves a sensing range of 11.2 m.

Table 3 compares the performance of different single ultrasonic transducers currently used for distance measurement. Utilizing a single transducer for long-distance ranging imposes significant demands on both the transmission and reception capabilities. The PMUT discussed in this paper exhibits exceptional output sound pressure and reception sensitivity, thereby achieving the longest measurement distance reported to date. This significant advancement facilitates the development of the PMUT for long-range detection applications. Additionally, for future applications that require even longer detection ranges, the PMUT can be implemented in arrays to further extend the sensing range.

## 7. Conclusions

In summary, we have introduced a ScAlN PMUT based on a flexurally suspended membrane. Compared to a traditional clamping PMUT operating at the same resonant frequency, our proposed design demonstrates significant improvements. Specifically, the output pressure is enhanced by approximately threefold and the effective coupling coefficient is increased by twofold. In distance testing, a single PMUT achieved a remarkable target detection capability of 11.2 m, surpassing the sensing range of any previously reported PMUT. Overall, our experiments validate the great potential of this PMUT for miniaturized and integrated long-range target detection applications.

## Figures and Tables

**Table 1:** Structural parameters used for the simulation of the FS-PMUT and T-PMUT.

Parameter	FS-PMUT	T-PMUT
Thickness of the top Au	200 nm	200 nm
Width of the groove	50 μm	-
Angle of the groove	70°	-
Thickness of the ScAlN	1 μm	1 μm
Thickness of the bottom Mo	200 nm	200 nm
Thickness of the top Si	5 μm	5 μm
Thickness of SiO_2_	200 nm	200 nm
Radius of the top Au	410 μm	500 μm
Radius of the cavity	590 μm	715 μm

**Table 2:** Comparison of the test results for three different PMUT designs.

Type	FS-PMUT (Sealed Groove)	FS-PMUT (Open Groove)	T-PMUT
Center displacement	3.83 μm	4.02 μm	2.25 μm
Effective vibration area	37.2%	38.3%	24%
Output pressure	112.2 dB	107.4 dB	102.8 dB
Reception sensitivity	12.3 mV/Pa	5.9 mV/Pa	8.6 mV/Pa
Effective coupling coefficient	4.45%	4%	2.37%

**Table 3:** Comparison of different single transducers for distance measurement.

References	Device Type	Piezoelectric Material	Frequency (kHz)	SPL (dB)	Max Range (m)
[42]	single	AlN	33	100	1.4
[8]	single	PZT	180	98	2
[43]	single	AlN	82	-	5
[25]	single	AlN	50	-	9
This work	single	ScAlN	78	112.2	11.2

## Data Availability

The original contributions presented in the study are included in the article, further inquiries can be directed to the corresponding author.
